# Dichloridobis{2-[(dimethyl­amino)­meth­yl]phen­yl}bis­{2-[(dimethyl­aza­nium­yl)meth­yl]phen­yl}di-μ-hydroxido-di-μ_3_-oxido-tetra­phenyl­tetra­tin(IV) dichloride deuterochloro­form deca­solvate

**DOI:** 10.1107/S1600536811048872

**Published:** 2011-11-30

**Authors:** Jan Turek, Zdeňka Padělková

**Affiliations:** a Department of General and Inorganic Chemistry, Faculty of Chemical Technology, University of Pardubice, Studentská 573, 53210 Pardubice, Czech Republic

## Abstract

The ladder-like structure of the tetranuclear title compound, [Sn_4_(C_6_H_5_)_4_Cl_2_O_2_(OH)_2_(C_9_H_13_N)_2_(C_9_H_12_N)_2_]Cl_2_·10CDCl_3_, consists of two five- and two six-coordinated Sn^IV^ atoms bridged by oxide or hydroxide groups. The chelating ligands reveal rather strong Sn—N bonds [2.517 (4) Å], but the protonated dimethylamino groups in the periphery of the complex show no interaction with the metal atoms. The complex cation is located on an inversion centre. The chloride anion is linked to the complex mol­ecule by strong intra­molecular O—H⋯Cl and N—H⋯Cl hydrogen bonds. Five independent deuterochloroform accompany the complex, two of them are disordered [occupancy ratios 0.63 (2):0.27 (2) and 0.60 (2):0.40 (2)].

## Related literature

For related structures, see: Novák *et al.* (2006 ▶, 2007 ▶); Varga & Silvestru (2007 ▶); Thoonen *et al.* (2006 ▶); Jambor *et al.* (2001 ▶); Padělková *et al.* (2007 ▶). For similar tetra­nuclear aggregates, see: Beckmann *et al.* (2001 ▶); Cox & Tiekink (1994 ▶); Kresinski *et al.* (1994 ▶); Lo & Ng (2009 ▶); Mohamed *et al.* (2004 ▶); Puff *et al.* (1983 ▶); Tiekink (1991 ▶); Vollano *et al.* (1984 ▶); Zhang *et al.* (2009 ▶). For similar hydrogen bonding in *C,N*-chelated organotin compounds, see: Padělková *et al.* (2009 ▶); Švec *et al.* (2010 ▶, 2011 ▶). 
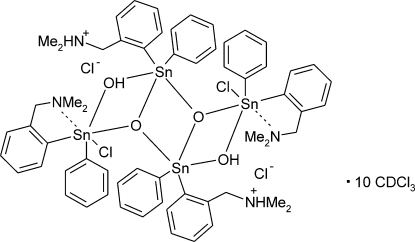


## Experimental

### 

#### Crystal data

[Sn_4_(C_6_H_5_)_4_Cl_2_O_2_(OH)_2_(C_9_H_13_N)_2_(C_9_H_12_N)_2_]Cl_2_·10CDCl_3_
*M* *_r_* = 2733.56
Triclinic, 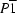

*a* = 11.9279 (5) Å
*b* = 15.4080 (12) Å
*c* = 15.9651 (14) Å
α = 83.752 (7)°
β = 68.178 (4)°
γ = 76.339 (5)°
*V* = 2646.1 (3) Å^3^
*Z* = 1
Mo *K*α radiation
μ = 1.84 mm^−1^
*T* = 150 K
0.28 × 0.26 × 0.21 mm

#### Data collection

Bruker–Nonius KappaCCD area-detector diffractometer
Absorption correction: gaussian (Coppens, 1970 ▶) *T*_min_ = 0.758, *T*_max_ = 0.820
51386 measured reflections
12052 independent reflections
9459 reflections with *I* > 2σ(*I*)
*R*_int_ = 0.065

#### Refinement

*R*[*F* ^2^ > 2σ(*F* ^2^)] = 0.054
*wR*(*F* ^2^) = 0.146
*S* = 1.08
12052 reflections
598 parameters
12 restraints
H-atom parameters constrained
Δρ_max_ = 1.95 e Å^−3^
Δρ_min_ = −1.15 e Å^−3^

### 

Data collection: *COLLECT* (Hooft, 1998 ▶) and *DENZO* (Otwinowski & Minor, 1997 ▶); cell refinement: *COLLECT* and *DENZO*; data reduction: *COLLECT* and *DENZO*; program(s) used to solve structure: *SHELXS97* (Sheldrick, 2008 ▶); program(s) used to refine structure: *SHELXL97* (Sheldrick, 2008 ▶); molecular graphics: *PLATON* (Spek, 2009 ▶); software used to prepare material for publication: *SHELXL97*.

## Supplementary Material

Crystal structure: contains datablock(s) I, global. DOI: 10.1107/S1600536811048872/aa2022sup1.cif

Structure factors: contains datablock(s) I. DOI: 10.1107/S1600536811048872/aa2022Isup2.hkl

Additional supplementary materials:  crystallographic information; 3D view; checkCIF report

## Figures and Tables

**Table 1:** Hydrogen-bond geometry (Å, °)

*D*—H⋯*A*	*D*—H	H⋯*A*	*D*⋯*A*	*D*—H⋯*A*
O2—H2⋯Cl2	0.93	2.21	3.079 (4)	156.0
N1—H1⋯Cl2	0.91	2.30	3.106 (5)	147.6

**Fig. 1.**
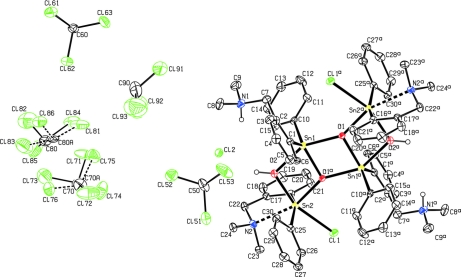
View of the title compound with the displacement ellipsoids shown on 50% probability level. Most H atoms are omitted for clarity, H atoms involved in hydrogen bonding are shown. Symmetry code: (a) ?x + 2, ?y + 2, ?z.

**Fig. 2.**
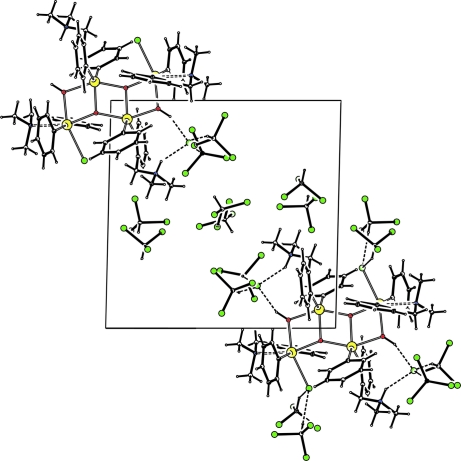
View of the I structure with the hydrogen bonding along the a axis.

## supplementary crystallographic information

### Comment

The common feature of the interaction of *C,N*-chelated organotin(IV) compounds with protic acids, as well as the mono- and diorganotin compounds, which have the most Lewis acidic tin atom centre, is the hydrolysis. A plethora of such hydrolytic products was identified in the past, including a number of molecular structures determined by X-ray diffraction techniques. The molecular structure of the title compound (Fig. 1) consists of a tetranuclear, nearly planar, aggregate of two central L^CN^PhSnO units and two peripherical L^CN^PhSn(OH)Cl units (L^CN^ = 2-(Me_2_NCH_2_)C_6_H_4_-). Similar tetranuclear aggregates were found earlier, for example for octabenzyl- (Mohamed *et al.*, 2004), octaisopropyl- (Puff *et al.*, 1983), octa(trimethylsilylmethyl)- (Puff *et al.*, 1983), octaphenyl- (Vollano *et al.*, 1984), octa(2-chlorobenzyl)- (Zhang *et al.*, 2009) , octa(4-chlorobenzyl)- (Lo & Ng, 2009) octaphenyl (Kresinski *et al*, 1994; Cox & Tiekink, 1994; Tiekink, 1991) and tetra(trimethylsilylmethyl)-tetra-t-butyl- (Beckmann *et al.*, 2001) derivative, respectively. The major difference between I and the rest of compounds is that all these compounds have all the tin atoms five coordinated. In I, the chelating dimethylaminomethyl arms of outer units are protonated by HCl. The chlorine anion is being out of the primary tin coordination sphere but is strongly connected by hydrogen bridges to both OH and NH groups (3.079 (4) Å, 3.106 (5) Å; Fig. 2). These hydrogen bonds are typical for *C,N*-chelated organotin compounds, as for example for L^CN^(*n*-Bu)_2_SnCl.HCl (Švec *et al.*, 2010) or [L^CN^(*n*-Bu)SnOC(O)CF_3_]_2_(µ-OH)_2_ (Švec *et al.*, 2011), but the presence of both types of hydrogen bonds in the same molecule were not observed before. The inner tin atoms coordination polyhedra are of very deformed trigonal bipyramids with both carbon atoms of the ligand and phenyl substituent and the bridging oxygen atom in equatorial positions, despite of that the value of the sum of interatomic angles in equatorial girdle is rather low (323.7°). The axial positions of these tin atoms are filled with the bridging OH group and the second bridging oxygen atom, where Sn1–O2 distances are a bit longer than in cases of the equatorial ones. The equatorial angle is shorter than ideally 180° being only 150.66 (13)°. The outer tin atoms have the *C,C*-transoidal coordination geometry of strongly deformed octahedron with mutually *cis*-coordinated O and OH groups, and chlorine and strongly interacting amino nitrogen (Sn–N 2.517 (4) Å) atoms, respectively. The C16–Sn2–C25 angle is wider (158.41 (19)°) than similar angles found in related ladder-like structures (~ 125°, for references see above). The solvent molecules (deuterochloroform) interact with the chloride anions.

### Experimental

The title compound (I, Scheme 1) has been obtained from the reaction mixture of ethylacetoacetate, cyclohexanol and [L^CN^PhSn(Cl)]_2_O (L^CN^ = 2-(Me_2_NCH_2_)C_6_H_4_-) (1 mol %) in deuterochloroform used as a catalyst in this transesterfication reaction (Padělková *et al.*, 2009) by slow evaporation on the air.

### Refinement

All the hydrogens were discernible in the difference electron density map. However, all the hydrogens were situated into idealized positions and refined riding on their parent C, O or N atoms, with O–H = 0.82 Å, N–H = 0.86 Å, C–H = 0.97 Å for methylene, 0.96 Å for methine, 0.93 Å for aromatic H atoms, with U(H) = 1.5U*_eq_*(C/O) for methyl and OH groups and U(H) = 1.2U*_eq_*(C/N) for other H atoms. SHELXL ISOR restraints were applied to the C atoms of the disordered deuterochloroform molecules.

other H atoms, respectively. There is disordered solvent (chlororform) in this structure. Successful attempts were made to resolve the disorder.

### Crystal data

**Table:**

[Sn_4_(C_6_H_5_)_4_Cl_2_O_2_(OH)_2_(C_9_H_13_N)_2_(C_9_H_12_N)_2_]Cl_2_·10CDCl_3_	*Z* = 1
*M**_r_* = 2733.56	*F*(000) = 1340
Triclinic, *P*1	*D*_x_ = 1.715 Mg m^−^^3^
Hall symbol: -P 1	Melting point: 395(2) K
*a* = 11.9279 (5) Å	Mo *K*α radiation, λ = 0.71073 Å
*b* = 15.4080 (12) Å	Cell parameters from 51386 reflections
*c* = 15.9651 (14) Å	θ = 1–27.5°
α = 83.752 (7)°	µ = 1.84 mm^−^^1^
β = 68.178 (4)°	*T* = 150 K
γ = 76.339 (5)°	Block, colourless
*V* = 2646.1 (3) Å^3^	0.28 × 0.26 × 0.21 mm

### Data collection

**Table:**

Bruker–Nonius KappaCCD area-detector diffractometer	12052 independent reflections
Radiation source: fine-focus sealed tube	9459 reflections with *I* > 2σ(*I*)
graphite	*R*_int_ = 0.065
Detector resolution: 9.091 pixels mm^-1^	θ_max_ = 27.5°, θ_min_ = 1.9°
φ and ω scans to fill the Ewald sphere	*h* = −14→15
Absorption correction: gaussian (Coppens, 1970)	*k* = −19→20
*T*_min_ = 0.758, *T*_max_ = 0.820	*l* = 0→20
51386 measured reflections	

### Refinement

**Table:**

Refinement on *F*^2^	Primary atom site location: structure-invariant direct methods
Least-squares matrix: full	Secondary atom site location: difference Fourier map
*R*[*F*^2^ > 2σ(*F*^2^)] = 0.054	Hydrogen site location: inferred from neighbouring sites
*wR*(*F*^2^) = 0.146	H-atom parameters constrained
*S* = 1.08	*w* = 1/[σ^2^(*F*_o_^2^) + (0.0647*P*)^2^ + 12.7718*P*] where *P* = (*F*_o_^2^ + 2*F*_c_^2^)/3
12052 reflections	(Δ/σ)_max_ = 0.001
598 parameters	Δρ_max_ = 1.95 e Å^−^^3^
12 restraints	Δρ_min_ = −1.15 e Å^−^^3^

### Special details

**Table:**

Geometry. All esds (except the esd in the dihedral angle between two l.s. planes) are estimated using the full covariance matrix. The cell esds are taken into account individually in the estimation of esds in distances, angles and torsion angles; correlations between esds in cell parameters are only used when they are defined by crystal symmetry. An approximate (isotropic) treatment of cell esds is used for estimating esds involving l.s. planes.
Refinement. Refinement of F^2^ against ALL reflections. The weighted R-factor wR and goodness of fit S are based on F^2^, conventional R-factors R are based on F, with F set to zero for negative F^2^. The threshold expression of F^2^ > 2sigma(F^2^) is used only for calculating R-factors(gt) etc. and is not relevant to the choice of reflections for refinement. R-factors based on F^2^ are statistically about twice as large as those based on F, and R- factors based on ALL data will be even larger.

### Fractional atomic coordinates and isotropic or equivalent isotropic displacement parameters (Å^2^)

**Table:**

	*x*	*y*	*z*	*U*_iso_*/*U*_eq_	Occ. (<1)
Sn1	0.95841 (3)	0.92734 (2)	0.08172 (2)	0.01807 (9)	
Sn2	1.05036 (3)	0.81140 (2)	−0.10648 (2)	0.01954 (9)	
Cl2	0.90846 (13)	0.65544 (9)	0.18634 (10)	0.0339 (3)	
Cl1	1.09431 (12)	0.89042 (9)	−0.26447 (8)	0.0299 (3)	
O2	0.9965 (3)	0.7941 (2)	0.0363 (2)	0.0231 (7)	
H2	0.9909	0.7414	0.0705	0.035*	
O1	0.9676 (3)	1.0635 (2)	0.0551 (2)	0.0197 (7)	
C17	0.8196 (5)	0.7435 (3)	−0.0818 (4)	0.0262 (11)	
N1	0.8708 (5)	0.7981 (3)	0.3231 (3)	0.0329 (10)	
H1	0.8901	0.7755	0.2679	0.039*	
N2	1.0295 (4)	0.6531 (3)	−0.1091 (3)	0.0262 (9)	
C6	1.2090 (5)	0.8765 (3)	0.0998 (4)	0.0257 (11)	
H6	1.2325	0.8850	0.0376	0.031*	
C21	0.7933 (5)	0.9018 (4)	−0.1142 (4)	0.0305 (12)	
H21	0.8256	0.9531	−0.1293	0.037*	
C24	1.1034 (5)	0.5831 (4)	−0.0692 (4)	0.0317 (12)	
H24A	1.1897	0.5821	−0.1010	0.048*	
H24B	1.0868	0.5262	−0.0735	0.048*	
H24C	1.0821	0.5956	−0.0068	0.048*	
C1	1.0852 (5)	0.8880 (3)	0.1516 (4)	0.0230 (10)	
C25	1.2430 (4)	0.7559 (3)	−0.1350 (4)	0.0232 (10)	
C10	0.7635 (4)	0.9427 (3)	0.1424 (3)	0.0238 (10)	
C16	0.8670 (5)	0.8216 (3)	−0.1023 (4)	0.0246 (10)	
C2	1.0492 (5)	0.8764 (4)	0.2462 (4)	0.0267 (11)	
C30	1.2839 (5)	0.7242 (4)	−0.0643 (4)	0.0285 (11)	
H30	1.2272	0.7265	−0.0055	0.034*	
C3	1.1427 (5)	0.8541 (4)	0.2844 (4)	0.0325 (12)	
H3	1.1204	0.8475	0.3467	0.039*	
C27	1.4542 (5)	0.7151 (4)	−0.2384 (4)	0.0353 (13)	
H27	1.5114	0.7126	−0.2971	0.042*	
C5	1.2996 (5)	0.8526 (4)	0.1379 (4)	0.0331 (12)	
H5	1.3828	0.8437	0.1016	0.040*	
C29	1.4068 (5)	0.6895 (4)	−0.0799 (4)	0.0337 (12)	
H29	1.4329	0.6692	−0.0318	0.040*	
C22	0.8976 (5)	0.6565 (4)	−0.0624 (4)	0.0289 (11)	
H22A	0.8800	0.6498	0.0021	0.035*	
H22B	0.8757	0.6071	−0.0812	0.035*	
C26	1.3293 (5)	0.7499 (4)	−0.2228 (4)	0.0318 (12)	
H26	1.3035	0.7693	−0.2712	0.038*	
C15	0.7063 (5)	0.8753 (4)	0.1408 (4)	0.0316 (12)	
H15	0.7531	0.8228	0.1111	0.038*	
C7	0.9177 (5)	0.8830 (4)	0.3073 (4)	0.0314 (12)	
H7A	0.8659	0.9279	0.2823	0.038*	
H7B	0.9081	0.9039	0.3652	0.038*	
C18	0.6992 (5)	0.7496 (4)	−0.0742 (4)	0.0340 (13)	
H18	0.6671	0.6983	−0.0615	0.041*	
C23	1.0672 (6)	0.6345 (4)	−0.2063 (4)	0.0354 (13)	
H23A	1.1539	0.6329	−0.2359	0.053*	
H23B	1.0214	0.6808	−0.2336	0.053*	
H23C	1.0505	0.5779	−0.2122	0.053*	
C4	1.2641 (6)	0.8420 (4)	0.2317 (4)	0.0352 (13)	
H4	1.3239	0.8267	0.2580	0.042*	
C28	1.4927 (5)	0.6847 (4)	−0.1682 (5)	0.0374 (14)	
H28	1.5759	0.6609	−0.1789	0.045*	
C19	0.6255 (5)	0.8298 (4)	−0.0850 (5)	0.0388 (14)	
H19	0.5444	0.8326	−0.0798	0.047*	
C20	0.6726 (6)	0.9066 (4)	−0.1038 (5)	0.0397 (14)	
H20	0.6224	0.9615	−0.1093	0.048*	
C14	0.5783 (5)	0.8856 (4)	0.1835 (4)	0.0352 (13)	
H14	0.5403	0.8397	0.1824	0.042*	
C11	0.6917 (5)	1.0208 (4)	0.1856 (4)	0.0354 (14)	
H11	0.7292	1.0671	0.1863	0.042*	
C13	0.5093 (5)	0.9624 (4)	0.2265 (5)	0.0415 (15)	
H13	0.4244	0.9686	0.2551	0.050*	
C9	0.7376 (6)	0.8137 (5)	0.3664 (5)	0.0482 (17)	
H9A	0.6998	0.8582	0.3325	0.072*	
H9B	0.7140	0.8340	0.4266	0.072*	
H9C	0.7108	0.7591	0.3689	0.072*	
C12	0.5639 (6)	1.0314 (4)	0.2281 (6)	0.0514 (19)	
H12	0.5163	1.0841	0.2570	0.062*	
C8	0.9315 (7)	0.7289 (5)	0.3745 (5)	0.0475 (17)	
H8A	1.0196	0.7192	0.3448	0.071*	
H8B	0.9051	0.6741	0.3771	0.071*	
H8C	0.9089	0.7489	0.4346	0.071*	
C50	1.2065 (7)	0.5493 (5)	0.1665 (7)	0.058 (2)	
D50	1.1290	0.5764	0.1569	0.070*	
Cl51	1.3126 (2)	0.50334 (13)	0.06538 (15)	0.0599 (5)	
Cl52	1.1760 (2)	0.46445 (16)	0.25107 (16)	0.0680 (6)	
Cl53	1.2564 (3)	0.6331 (2)	0.1980 (3)	0.1126 (13)	
C60	0.3786 (6)	0.4030 (5)	0.7682 (5)	0.0476 (16)	
D60	0.2932	0.4094	0.7718	0.057*	
Cl61	0.3904 (2)	0.35616 (16)	0.87085 (14)	0.0653 (5)	
Cl62	0.4761 (2)	0.33095 (14)	0.68003 (14)	0.0679 (6)	
Cl63	0.4152 (2)	0.50854 (13)	0.74811 (19)	0.0746 (7)	
C70A	1.141 (4)	0.165 (3)	0.361 (3)	0.089 (13)	0.37 (2)
D70A	1.1293	0.1533	0.3064	0.107*	0.37 (2)
Cl75	0.992 (2)	0.2562 (10)	0.4102 (13)	0.093 (5)	0.37 (2)
Cl74	1.217 (2)	0.2208 (11)	0.3248 (8)	0.107 (5)	0.37 (2)
Cl76	1.1541 (16)	0.0616 (7)	0.4047 (10)	0.094 (4)	0.37 (2)
C70	1.0976 (12)	0.1618 (10)	0.3631 (8)	0.038 (3)	0.63 (2)
D70	1.0899	0.1513	0.3063	0.045*	0.63 (2)
Cl71	0.9801 (12)	0.2362 (10)	0.4232 (8)	0.141 (5)	0.63 (2)
Cl72	1.2500 (6)	0.1749 (11)	0.3461 (8)	0.137 (5)	0.63 (2)
Cl73	1.0761 (16)	0.0688 (5)	0.4391 (7)	0.119 (4)	0.63 (2)
C80	0.670 (3)	0.167 (2)	0.4596 (17)	0.049 (8)	0.40 (2)
D80	0.7492	0.1555	0.4089	0.059*	0.40 (2)
Cl81	0.619 (2)	0.2718 (12)	0.4575 (9)	0.108 (5)	0.40 (2)
Cl82	0.7067 (8)	0.1475 (7)	0.5587 (5)	0.066 (3)	0.40 (2)
Cl83	0.5908 (14)	0.0680 (14)	0.4646 (18)	0.133 (7)	0.40 (2)
C80A	0.652 (3)	0.1390 (16)	0.4638 (19)	0.064 (7)	0.60 (2)
D80A	0.7375	0.1329	0.4210	0.077*	0.60 (2)
Cl84	0.5572 (11)	0.2548 (6)	0.4667 (4)	0.095 (3)	0.60 (2)
Cl86	0.6429 (19)	0.1145 (11)	0.5677 (6)	0.159 (7)	0.60 (2)
Cl85	0.5654 (14)	0.0996 (8)	0.4272 (7)	0.143 (4)	0.60 (2)
C90	0.7388 (15)	0.4945 (8)	0.5226 (12)	0.124 (5)	
D90	0.6980	0.4604	0.5763	0.148*	
Cl91	0.7081 (5)	0.6026 (3)	0.5531 (3)	0.169 (2)	
Cl92	0.9047 (7)	0.4615 (5)	0.4986 (5)	0.220 (3)	
Cl93	0.6965 (8)	0.4755 (5)	0.4449 (6)	0.226 (3)	

### Atomic displacement parameters (Å^2^)

**Table:**

	*U*^11^	*U*^22^	*U*^33^	*U*^12^	*U*^13^	*U*^23^
Sn1	0.01544 (16)	0.02144 (17)	0.01780 (16)	−0.00579 (12)	−0.00581 (12)	0.00129 (12)
Sn2	0.01716 (17)	0.02137 (17)	0.02117 (17)	−0.00468 (12)	−0.00731 (13)	−0.00201 (13)
Cl2	0.0337 (7)	0.0332 (7)	0.0328 (7)	−0.0078 (6)	−0.0101 (6)	0.0028 (6)
Cl1	0.0285 (6)	0.0367 (7)	0.0217 (6)	−0.0045 (5)	−0.0079 (5)	0.0009 (5)
O2	0.0244 (18)	0.0201 (17)	0.0257 (18)	−0.0057 (14)	−0.0100 (15)	0.0023 (14)
O1	0.0223 (17)	0.0207 (16)	0.0160 (15)	−0.0067 (13)	−0.0057 (13)	0.0001 (12)
C17	0.026 (3)	0.025 (3)	0.030 (3)	−0.006 (2)	−0.011 (2)	−0.005 (2)
N1	0.037 (3)	0.040 (3)	0.024 (2)	−0.013 (2)	−0.011 (2)	0.002 (2)
N2	0.029 (2)	0.024 (2)	0.028 (2)	−0.0054 (18)	−0.0125 (19)	−0.0030 (18)
C6	0.029 (3)	0.028 (3)	0.021 (2)	−0.009 (2)	−0.007 (2)	−0.003 (2)
C21	0.031 (3)	0.028 (3)	0.039 (3)	−0.009 (2)	−0.018 (2)	−0.001 (2)
C24	0.029 (3)	0.024 (3)	0.044 (3)	−0.007 (2)	−0.014 (3)	0.001 (2)
C1	0.025 (2)	0.022 (2)	0.028 (3)	−0.0080 (19)	−0.013 (2)	−0.002 (2)
C25	0.018 (2)	0.023 (2)	0.031 (3)	−0.0039 (18)	−0.010 (2)	−0.003 (2)
C10	0.016 (2)	0.028 (3)	0.024 (2)	−0.0062 (19)	−0.0041 (19)	0.006 (2)
C16	0.022 (2)	0.027 (3)	0.027 (3)	−0.008 (2)	−0.011 (2)	0.000 (2)
C2	0.027 (3)	0.027 (3)	0.030 (3)	−0.008 (2)	−0.014 (2)	0.001 (2)
C30	0.027 (3)	0.032 (3)	0.028 (3)	−0.010 (2)	−0.010 (2)	0.001 (2)
C3	0.034 (3)	0.042 (3)	0.026 (3)	−0.010 (2)	−0.016 (2)	0.002 (2)
C27	0.024 (3)	0.038 (3)	0.037 (3)	−0.006 (2)	−0.001 (2)	−0.008 (3)
C5	0.021 (3)	0.034 (3)	0.043 (3)	−0.005 (2)	−0.010 (2)	−0.004 (2)
C29	0.027 (3)	0.038 (3)	0.038 (3)	−0.003 (2)	−0.017 (2)	0.001 (2)
C22	0.028 (3)	0.024 (3)	0.041 (3)	−0.009 (2)	−0.017 (2)	0.002 (2)
C26	0.024 (3)	0.034 (3)	0.033 (3)	0.000 (2)	−0.009 (2)	−0.004 (2)
C15	0.020 (3)	0.031 (3)	0.042 (3)	−0.005 (2)	−0.009 (2)	−0.006 (2)
C7	0.032 (3)	0.038 (3)	0.026 (3)	−0.008 (2)	−0.012 (2)	0.000 (2)
C18	0.031 (3)	0.034 (3)	0.046 (3)	−0.016 (2)	−0.020 (3)	0.002 (3)
C23	0.043 (3)	0.036 (3)	0.027 (3)	−0.007 (3)	−0.011 (3)	−0.007 (2)
C4	0.035 (3)	0.040 (3)	0.039 (3)	−0.009 (3)	−0.024 (3)	0.003 (3)
C28	0.020 (3)	0.039 (3)	0.052 (4)	−0.001 (2)	−0.014 (3)	−0.009 (3)
C19	0.025 (3)	0.039 (3)	0.061 (4)	−0.009 (2)	−0.022 (3)	−0.005 (3)
C20	0.029 (3)	0.031 (3)	0.062 (4)	−0.002 (2)	−0.023 (3)	−0.003 (3)
C14	0.022 (3)	0.036 (3)	0.048 (4)	−0.010 (2)	−0.009 (3)	−0.005 (3)
C11	0.019 (3)	0.029 (3)	0.051 (4)	−0.010 (2)	0.000 (2)	−0.010 (3)
C13	0.017 (3)	0.046 (4)	0.053 (4)	−0.009 (2)	−0.004 (3)	0.007 (3)
C9	0.042 (4)	0.059 (4)	0.041 (4)	−0.021 (3)	−0.005 (3)	−0.003 (3)
C12	0.026 (3)	0.033 (3)	0.083 (5)	0.002 (3)	−0.007 (3)	−0.019 (3)
C8	0.063 (5)	0.046 (4)	0.042 (4)	−0.023 (3)	−0.025 (3)	0.018 (3)
C50	0.038 (4)	0.038 (4)	0.104 (7)	−0.004 (3)	−0.033 (4)	−0.007 (4)
Cl51	0.0641 (12)	0.0436 (9)	0.0749 (13)	−0.0156 (9)	−0.0282 (10)	0.0075 (9)
Cl52	0.0540 (11)	0.0742 (14)	0.0687 (13)	−0.0092 (10)	−0.0166 (10)	−0.0027 (11)
Cl53	0.0833 (18)	0.0798 (17)	0.175 (3)	−0.0312 (14)	−0.0204 (19)	−0.066 (2)
C60	0.037 (3)	0.050 (4)	0.050 (4)	−0.011 (3)	−0.005 (3)	−0.011 (3)
Cl61	0.0648 (13)	0.0799 (14)	0.0492 (11)	−0.0178 (11)	−0.0140 (9)	−0.0108 (10)
Cl62	0.0832 (15)	0.0562 (11)	0.0487 (11)	−0.0074 (10)	−0.0056 (10)	−0.0183 (9)
Cl63	0.0578 (12)	0.0408 (10)	0.1034 (18)	−0.0133 (9)	0.0011 (12)	−0.0153 (11)
C70A	0.093 (16)	0.078 (15)	0.087 (15)	−0.010 (10)	−0.025 (10)	−0.005 (9)
Cl75	0.126 (11)	0.062 (5)	0.080 (7)	0.011 (5)	−0.045 (6)	0.001 (5)
Cl74	0.154 (12)	0.120 (9)	0.094 (6)	−0.090 (9)	−0.070 (7)	0.038 (6)
Cl76	0.111 (9)	0.056 (4)	0.092 (7)	0.001 (5)	−0.026 (6)	0.014 (4)
C70	0.030 (6)	0.060 (7)	0.031 (5)	−0.006 (5)	−0.020 (5)	−0.008 (5)
Cl71	0.118 (7)	0.197 (10)	0.107 (6)	0.084 (7)	−0.083 (6)	−0.105 (7)
Cl72	0.062 (3)	0.223 (11)	0.115 (7)	0.007 (5)	−0.024 (3)	−0.065 (7)
Cl73	0.184 (11)	0.089 (4)	0.092 (5)	−0.005 (5)	−0.074 (7)	−0.001 (3)
C80	0.066 (18)	0.051 (16)	0.016 (8)	0.006 (13)	−0.008 (9)	−0.005 (9)
Cl81	0.088 (9)	0.144 (9)	0.064 (5)	0.038 (8)	−0.020 (6)	−0.048 (5)
Cl82	0.059 (5)	0.094 (5)	0.045 (3)	0.008 (3)	−0.032 (3)	−0.010 (3)
Cl83	0.118 (8)	0.109 (11)	0.156 (16)	−0.028 (8)	−0.019 (9)	−0.043 (10)
C80A	0.047 (8)	0.064 (11)	0.063 (9)	0.003 (7)	−0.007 (6)	−0.004 (7)
Cl84	0.078 (5)	0.121 (5)	0.049 (2)	0.027 (4)	−0.008 (3)	−0.013 (3)
Cl86	0.177 (13)	0.175 (10)	0.097 (5)	0.037 (10)	−0.063 (6)	0.006 (5)
Cl85	0.143 (8)	0.132 (7)	0.107 (6)	−0.035 (6)	−0.002 (5)	0.041 (5)
C90	0.134 (13)	0.068 (7)	0.140 (13)	−0.014 (8)	−0.024 (10)	0.014 (8)
Cl91	0.195 (5)	0.098 (3)	0.116 (3)	0.013 (3)	0.028 (3)	0.004 (2)
Cl92	0.197 (6)	0.222 (7)	0.193 (6)	0.037 (5)	−0.058 (5)	−0.032 (5)
Cl93	0.252 (8)	0.238 (8)	0.219 (7)	−0.082 (6)	−0.102 (7)	−0.001 (6)

### Geometric parameters (Å, °)

**Table:**

Sn1—O1^i^	2.031 (3)	C15—H15	0.9300
Sn1—O1	2.115 (3)	C7—H7A	0.9700
Sn1—C10	2.125 (5)	C7—H7B	0.9700
Sn1—C1	2.139 (5)	C18—C19	1.372 (9)
Sn1—O2	2.142 (3)	C18—H18	0.9300
Sn1—Sn1^i^	3.2649 (7)	C23—H23A	0.9600
Sn2—O1^i^	2.110 (3)	C23—H23B	0.9600
Sn2—O2	2.130 (4)	C23—H23C	0.9600
Sn2—C16	2.132 (5)	C4—H4	0.9300
Sn2—C25	2.144 (5)	C28—H28	0.9300
Sn2—N2	2.517 (4)	C19—C20	1.385 (9)
Sn2—Cl1	2.6017 (13)	C19—H19	0.9300
O2—H2	0.9300	C20—H20	0.9300
O1—Sn1^i^	2.031 (3)	C14—C13	1.359 (9)
O1—Sn2^i^	2.110 (3)	C14—H14	0.9300
C17—C18	1.377 (8)	C11—C12	1.396 (8)
C17—C16	1.404 (7)	C11—H11	0.9300
C17—C22	1.515 (7)	C13—C12	1.377 (9)
N1—C9	1.448 (8)	C13—H13	0.9300
N1—C8	1.490 (8)	C9—H9A	0.9600
N1—C7	1.502 (8)	C9—H9B	0.9600
N1—H1	0.9100	C9—H9C	0.9600
N2—C22	1.460 (7)	C12—H12	0.9300
N2—C24	1.472 (7)	C8—H8A	0.9600
N2—C23	1.486 (7)	C8—H8B	0.9600
C6—C1	1.377 (7)	C8—H8C	0.9600
C6—C5	1.387 (8)	C50—Cl53	1.732 (8)
C6—H6	0.9300	C50—Cl51	1.737 (9)
C21—C20	1.371 (8)	C50—Cl52	1.772 (9)
C21—C16	1.377 (8)	C50—D50	0.9800
C21—H21	0.9300	C60—Cl63	1.748 (7)
C24—H24A	0.9600	C60—Cl62	1.754 (7)
C24—H24B	0.9600	C60—Cl61	1.759 (8)
C24—H24C	0.9600	C60—D60	0.9800
C1—C2	1.411 (7)	C70A—Cl74	1.32 (5)
C25—C30	1.388 (8)	C70A—Cl76	1.67 (4)
C25—C26	1.395 (8)	C70A—Cl75	1.94 (5)
C10—C15	1.376 (7)	C70A—D70A	0.9800
C10—C11	1.383 (8)	Cl75—Cl74	2.47 (3)
C2—C3	1.418 (8)	C70—Cl71	1.636 (17)
C2—C7	1.494 (8)	C70—Cl73	1.776 (17)
C30—C29	1.373 (8)	C70—Cl72	1.793 (14)
C30—H30	0.9300	C70—D70	0.9800
C3—C4	1.356 (9)	C80—Cl81	1.58 (3)
C3—H3	0.9300	C80—Cl82	1.77 (3)
C27—C28	1.363 (9)	C80—Cl83	1.96 (4)
C27—C26	1.394 (8)	C80—D80	0.9800
C27—H27	0.9300	C80A—Cl85	1.61 (3)
C5—C4	1.398 (9)	C80A—Cl86	1.63 (3)
C5—H5	0.9300	C80A—Cl84	1.87 (3)
C29—C28	1.398 (9)	C80A—D80A	0.9800
C29—H29	0.9300	C90—Cl93	1.579 (18)
C22—H22A	0.9700	C90—Cl91	1.704 (14)
C22—H22B	0.9700	C90—Cl92	1.824 (17)
C26—H26	0.9300	C90—D90	0.9800
C15—C14	1.400 (8)		
			
O1^i^—Sn1—O1	76.15 (14)	C27—C26—H26	119.8
O1^i^—Sn1—C10	117.58 (17)	C25—C26—H26	119.8
O1—Sn1—C10	98.97 (17)	C10—C15—C14	120.4 (5)
O1^i^—Sn1—C1	116.88 (17)	C10—C15—H15	119.8
O1—Sn1—C1	99.62 (16)	C14—C15—H15	119.8
C10—Sn1—C1	125.1 (2)	C2—C7—N1	115.8 (5)
O1^i^—Sn1—O2	74.63 (13)	C2—C7—H7A	108.3
O1—Sn1—O2	150.66 (13)	N1—C7—H7A	108.3
C10—Sn1—O2	96.77 (17)	C2—C7—H7B	108.3
C1—Sn1—O2	91.22 (16)	N1—C7—H7B	108.3
O1^i^—Sn1—Sn1^i^	38.98 (9)	H7A—C7—H7B	107.4
O1—Sn1—Sn1^i^	37.17 (9)	C19—C18—C17	121.5 (5)
C10—Sn1—Sn1^i^	112.90 (14)	C19—C18—H18	119.3
C1—Sn1—Sn1^i^	112.93 (14)	C17—C18—H18	119.3
O2—Sn1—Sn1^i^	113.57 (10)	N2—C23—H23A	109.5
O1^i^—Sn2—O2	73.31 (13)	N2—C23—H23B	109.5
O1^i^—Sn2—C16	100.53 (17)	H23A—C23—H23B	109.5
O2—Sn2—C16	92.56 (17)	N2—C23—H23C	109.5
O1^i^—Sn2—C25	100.96 (16)	H23A—C23—H23C	109.5
O2—Sn2—C25	95.58 (17)	H23B—C23—H23C	109.5
C16—Sn2—C25	158.41 (19)	C3—C4—C5	120.3 (5)
O1^i^—Sn2—N2	159.45 (14)	C3—C4—H4	119.9
O2—Sn2—N2	86.82 (14)	C5—C4—H4	119.9
C16—Sn2—N2	74.65 (17)	C27—C28—C29	119.8 (5)
C25—Sn2—N2	85.85 (17)	C27—C28—H28	120.1
O1^i^—Sn2—Cl1	87.05 (9)	C29—C28—H28	120.1
O2—Sn2—Cl1	159.94 (10)	C18—C19—C20	119.5 (5)
C16—Sn2—Cl1	86.94 (15)	C18—C19—H19	120.2
C25—Sn2—Cl1	92.07 (15)	C20—C19—H19	120.2
N2—Sn2—Cl1	112.25 (11)	C21—C20—C19	120.0 (6)
Sn2—O2—Sn1	103.23 (14)	C21—C20—H20	120.0
Sn2—O2—H2	128.4	C19—C20—H20	120.0
Sn1—O2—H2	128.4	C13—C14—C15	120.1 (5)
Sn1^i^—O1—Sn2^i^	107.90 (15)	C13—C14—H14	120.0
Sn1^i^—O1—Sn1	103.85 (14)	C15—C14—H14	120.0
Sn2^i^—O1—Sn1	147.88 (17)	C10—C11—C12	121.1 (5)
C18—C17—C16	118.4 (5)	C10—C11—H11	119.5
C18—C17—C22	121.4 (5)	C12—C11—H11	119.5
C16—C17—C22	120.0 (5)	C14—C13—C12	120.8 (5)
C9—N1—C8	110.4 (5)	C14—C13—H13	119.6
C9—N1—C7	112.3 (5)	C12—C13—H13	119.6
C8—N1—C7	112.7 (5)	N1—C9—H9A	109.5
C9—N1—H1	107.0	N1—C9—H9B	109.5
C8—N1—H1	107.0	H9A—C9—H9B	109.5
C7—N1—H1	107.0	N1—C9—H9C	109.5
C22—N2—C24	111.0 (4)	H9A—C9—H9C	109.5
C22—N2—C23	110.8 (4)	H9B—C9—H9C	109.5
C24—N2—C23	108.6 (4)	C13—C12—C11	119.0 (6)
C22—N2—Sn2	104.1 (3)	C13—C12—H12	120.5
C24—N2—Sn2	117.3 (3)	C11—C12—H12	120.5
C23—N2—Sn2	104.8 (3)	N1—C8—H8A	109.5
C1—C6—C5	121.8 (5)	N1—C8—H8B	109.5
C1—C6—H6	119.1	H8A—C8—H8B	109.5
C5—C6—H6	119.1	N1—C8—H8C	109.5
C20—C21—C16	120.5 (5)	H8A—C8—H8C	109.5
C20—C21—H21	119.7	H8B—C8—H8C	109.5
C16—C21—H21	119.7	Cl53—C50—Cl51	110.9 (4)
N2—C24—H24A	109.5	Cl53—C50—Cl52	112.4 (5)
N2—C24—H24B	109.5	Cl51—C50—Cl52	110.2 (4)
H24A—C24—H24B	109.5	Cl53—C50—D50	107.7
N2—C24—H24C	109.5	Cl51—C50—D50	107.7
H24A—C24—H24C	109.5	Cl52—C50—D50	107.7
H24B—C24—H24C	109.5	Cl63—C60—Cl62	110.9 (4)
C6—C1—C2	119.2 (5)	Cl63—C60—Cl61	110.8 (4)
C6—C1—Sn1	116.7 (4)	Cl62—C60—Cl61	109.5 (4)
C2—C1—Sn1	124.1 (4)	Cl63—C60—D60	108.6
C30—C25—C26	118.4 (5)	Cl62—C60—D60	108.6
C30—C25—Sn2	119.4 (4)	Cl61—C60—D60	108.6
C26—C25—Sn2	122.1 (4)	Cl74—C70A—Cl76	136 (3)
C15—C10—C11	118.7 (5)	Cl74—C70A—Cl75	97 (3)
C15—C10—Sn1	121.1 (4)	Cl76—C70A—Cl75	121 (2)
C11—C10—Sn1	120.1 (4)	Cl74—C70A—D70A	98.1
C21—C16—C17	120.0 (5)	Cl76—C70A—D70A	98.1
C21—C16—Sn2	122.4 (4)	Cl75—C70A—D70A	98.1
C17—C16—Sn2	117.6 (4)	C70A—Cl75—Cl74	32.0 (13)
C1—C2—C3	118.4 (5)	C70A—Cl74—Cl75	51 (2)
C1—C2—C7	122.9 (5)	Cl71—C70—Cl73	98.6 (11)
C3—C2—C7	118.6 (5)	Cl71—C70—Cl72	117.9 (10)
C29—C30—C25	121.0 (5)	Cl73—C70—Cl72	104.5 (8)
C29—C30—H30	119.5	Cl71—C70—D70	111.6
C25—C30—H30	119.5	Cl73—C70—D70	111.6
C4—C3—C2	121.2 (5)	Cl72—C70—D70	111.6
C4—C3—H3	119.4	Cl81—C80—Cl82	104.2 (16)
C2—C3—H3	119.4	Cl81—C80—Cl83	130 (2)
C28—C27—C26	120.3 (6)	Cl82—C80—Cl83	101.9 (18)
C28—C27—H27	119.9	Cl81—C80—D80	106.1
C26—C27—H27	119.9	Cl82—C80—D80	106.1
C6—C5—C4	119.1 (5)	Cl83—C80—D80	106.1
C6—C5—H5	120.5	Cl85—C80A—Cl86	117.5 (15)
C4—C5—H5	120.5	Cl85—C80A—Cl84	91.9 (16)
C30—C29—C28	120.0 (6)	Cl86—C80A—Cl84	105.2 (14)
C30—C29—H29	120.0	Cl85—C80A—D80A	113.3
C28—C29—H29	120.0	Cl86—C80A—D80A	113.3
N2—C22—C17	111.9 (4)	Cl84—C80A—D80A	113.3
N2—C22—H22A	109.2	Cl93—C90—Cl91	117.6 (10)
C17—C22—H22A	109.2	Cl93—C90—Cl92	114.9 (10)
N2—C22—H22B	109.2	Cl91—C90—Cl92	101.5 (9)
C17—C22—H22B	109.2	Cl93—C90—D90	107.4
H22A—C22—H22B	107.9	Cl91—C90—D90	107.4
C27—C26—C25	120.4 (6)	Cl92—C90—D90	107.4

Symmetry codes: (i) −*x*+2, −*y*+2, −*z*.

### Hydrogen-bond geometry (Å, °)

**Table:**

*D*—H···*A*	*D*—H	H···*A*	*D*···*A*	*D*—H···*A*
O2—H2···Cl2	0.93	2.21	3.079 (4)	156.0
N1—H1···Cl2	0.91	2.30	3.106 (5)	147.6
